# Association between immune cells and breast cancer: A two-sample Mendelian randomization study

**DOI:** 10.1097/MD.0000000000045218

**Published:** 2025-10-10

**Authors:** Wenzi Luo, Duo Han, Chunhui Liu, Wenwen Luo, Dequan Pang, Jinxin Bai, Ning Zhai

**Affiliations:** a Department of Oncology Radiation Physics Technology Section, North China University of Science and Technology Affiliated Hospital, Tangshan, Hebei Province, China; b Department of Anesthesiology, Tangshan Workers’ Hospital, Tangshan, Hebei Province, China.

**Keywords:** breast cancer, circulating immune cells, genetics, Mendelian randomization

## Abstract

This study explored the potential causal relationship between circulating immune cells and breast cancer incidence through a 2-sample Mendelian randomization (MR) approach using inverse-variance weighting, Weighted Median and MR-Egger regression analyses. Pooled statistical datasets from publicly accessible genome-wide association studies on individuals of European ancestry (n = 563085) were used as a base resource for exposure variables. Meanwhile, breast cancer registry codes extracted from the UK Biobank (n = 6563)were used as outcome measures. A total of 132 single-nucleotide polymorphisms of genome-wide significance were selected as instrumental variables from genome-wide association studies focusing on circulating immune cells. The association between the CD4RA gene pair terminally differentiated CD4^+^ lymphocyte ratio and breast cancer risk was estimated by MR analysis. An odds ratio of 0.9809 (95% CI: 0.9668–0.9952) and a *P*-value of .0089 were obtained using the inverse-variance weighting method, indicating a statistically significant protective association. This protective effect was also consistently supported in weighted median and simple mode analyses, but did not show significance in weighted mode analyses. Cochran *Q* test and funnel plot assessment did not reveal significant heterogeneity or asymmetry, suggesting no directional pleiotropy. This suggests that circulating immune cells may play a protective role in breast carcinogenesis through the CD4RA gene. These findings may provide valuable insights for the development of risk prediction models and preventive strategies, and may also inform functional studies to elucidate underlying mechanisms and identify potential therapeutic targets.

## 1. Introduction

breast cancer (BC) is the most frequently diagnosed malignancy among women worldwide. According to GLOBOCAN 2020, it accounts for 24.5% of new cancer cases and 15.5% of all female cancer-related deaths.^[1]^ Incidence rates vary markedly by geography, ethnicity, and age group: the age-standardized incidence rate exceeds 80 per 100,000 in North America and Western Europe, but falls below 40 per 100,000 in parts of Africa and South Asia. Mortality is disproportionately higher among women of African descent, and incidence in women under 40 is steadily rising.^[2]^

Despite extensive investigation, the etiology of BC remains incompletely understood. Known risk factors – such as BRCA1/2 mutations, hormonal exposure, reproductive history, breast density, body mass index, smoking, alcohol consumption, and lifestyle – only partially explain disease risk.^[3,4]^ Intriguingly, some women develop the disease in the absence of known risk factors, while others with multiple risk factors remain unaffected. These inconsistencies underscore the limitations of current risk models.

Of particular concern is the increasing incidence of BC in young women, for which the underlying mechanisms are poorly defined. This highlights a pressing need to identify novel risk factors and elucidate molecular pathways that drive disease initiation, ultimately supporting improved risk prediction and prevention.^[5]^

Circulating immune cells are increasingly recognized as dynamic peripheral indicators of the tumor immune microenvironment (TME) and play critical roles in tumor initiation, progression, and metastasis. With advances in immunomonitoring technologies, peripheral blood immunophenotyping has emerged as a promising noninvasive approach for detecting systemic immune alterations associated with cancer.

In BC, shifts in the abundance and function of immune subsets – particularly CD8⁺ cytotoxic T lymphocytes, regulatory T cells (Tregs), natural killer (NK) cells, and myeloid-derived suppressor cells (MDSCs) – have been associated with tumor burden, molecular subtype, therapeutic response, and prognosis.^[6–8]^ These alterations often result in CD8⁺ T cell exhaustion, Treg expansion, and MDSC accumulation, fostering an immunosuppressive milieu that facilitates immune evasion.

The cancer immunoediting hypothesis posits that tumors evolve through elimination, equilibrium, and escape phases, aided by the upregulation of immune checkpoint molecules such as PD-L1.^[9,10]^ Therefore, comprehensive profiling of circulating immune phenotypes may offer mechanistic insights into TME remodeling and identify novel biomarkers and therapeutic targets for early detection and precision immunotherapy.

Mendelian randomization (MR) offers a powerful approach for causal inference by exploiting the random allocation of germline variants as proxies for modifiable exposures, mimicking the structure of randomized controlled trials.^[11]^ Previous studies, such as those by Zhixuan Wu et al^[8]^ and Wanxian Xu et al,^[12]^ have provided important evidence supporting causal associations between immune cell traits and BC risk using MR and meta-analysis frameworks. Building upon these findings, in this study, we employed MR to assess whether immune cell traits have a causal effect on BC risk, leveraging large-scale genome-wide association study (GWAS) data.

Instrumental variables (IV) – single-nucleotide polymorphisms (SNPs) strongly associated with immune phenotypes – were selected from publicly available datasets, satisfying the 3 key MR assumptions: relevance, independence, and exclusion restriction.^[11]^

To enhance the robustness of our findings, we applied a suite of MR methods: inverse-variance weighting (IVW), weighted median, and MR-Egger regression. These complementary approaches allow for statistical efficiency, robustness to invalid instruments, and sensitivity to directional pleiotropy.^[13,14]^ Our study builds upon prior MR-based investigations into the causal effects of immune phenotypes on BC, providing broader coverage and deeper insights that bridge immunogenetics and cancer epidemiology. The key contributions include: (1) establishing a causal link between circulating immune cell traits and BC risk; (2) applying a multi-method validation framework to enhance reliability; and (3) providing new insights for immunogenetic applications in early detection and precision prevention.

## 2. Materials and methods

### 2.1. Data sources and selection of genetic variants

We extracted and analyzed data from the MR-Base platform (http://www.mrbase.org/), which provides harmonized summary-level statistics from large-scale genome-wide association studies (GWAS), encompassing a broad spectrum of genetic variants and phenotypic traits. Immune cell phenotype data were obtained from the GWAS Catalog (accession numbers GCST90001391 to GCST90002121), comprising 731 immune-related traits.^[15]^ As genetic instruments for exposure, we utilized summary statistics from the Blood Cell Consortium (BCX), based on a GWAS of 563,085 individuals of European ancestry, covering approximately 22 million SNPs and imputed using the Sardinian reference panel.^[16,17]^ This exposure GWAS includes data from multiple cohorts, including the UK Biobank.

For the outcome, BC GWAS data were derived from a large meta-analysis comprising 90,969 cases and 195,636 controls of European ancestry. Among these, 6563 BC cases from the UK Biobank were identified using national cancer registry codes. Although there is minor sample overlap between the exposure and outcome datasets (because some UK Biobank participants are part of both the blood cell GWAS and the BC GWAS), this overlap represents only a small proportion of the total sample size and is unlikely to meaningfully bias the MR results.

IV were selected as SNPs strongly associated with immune phenotypes (*P* < 5 × 10⁻⁸). To ensure compliance with the 3 core assumptions of MR – relevance, independence, and exclusion restriction – we implemented a 2-step filtering strategy. First, SNPs with weak instrument strength were excluded based on genome-wide significance to preserve strong exposure relevance. Second, potential confounding was addressed by screening all candidate SNPs using PhenoScanner v2 (http://www.phenoscanner.medschl.cam.ac.uk/) to identify genome-wide associations with known confounders, including body mass index, smoking, alcohol use, hormone levels, metabolic traits, and inflammatory markers (*P* < 5 × 10⁻⁸). SNPs with evidence of such associations were removed.

The final set included 132 independent SNPs, supporting a valid causal pathway from immune phenotypes to BC risk. This rigorous selection minimized horizontal pleiotropy and strengthened the validity and robustness of causal inference.

### 2.2. Data analysis of MR

**Figure 1.**
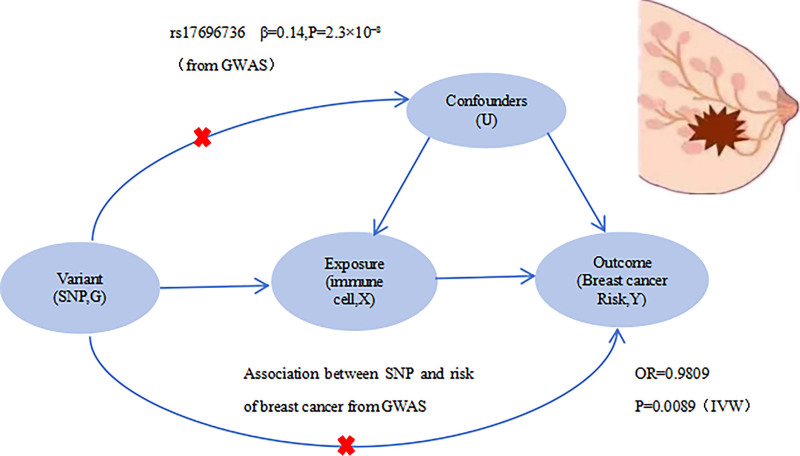
DAG of the Mendelian randomization framework. G: genetic variants (IV, e.g., SNPs); X: exposure (immune cell phenotype); U: unmeasured confounders (e.g., environmental or lifestyle factors); Y: outcome (BC risk). Arrows indicate the assumed causal pathways, and red crosses mark pathways that are blocked under the MR assumptions. BC = breast cancer, DAG = directed acyclic graph, IV = instrumental variables, GWAS = genome-wide association studies, MR = Mendelian randomization, SNP = single-nucleotide polymorphism.

To meet the core assumptions of MR, we selected 132 independent SNPs that demonstrated genome-wide significant associations with immune cell phenotypes and were not linked to known confounders (Fig. 1). These SNPs were used as IV in a 2-sample MR framework to estimate the causal effects of immune cell traits on BC risk.

IVW was employed as the primary method for causal estimation. This approach aggregates the Wald ratio estimates across SNPs to derive a combined causal effect, and is the most widely used and statistically powerful method in MR studies, provided that all instruments are valid and horizontal pleiotropy is absent.^[18]^ However, if some SNPs influence the outcome via pathways independent of the exposure (i.e., horizontal pleiotropy), IVW estimates may be biased. To address this, we incorporated MR-Egger regression, which accounts for directional pleiotropy by estimating an intercept term that reflects the average pleiotropic effect across instruments.

To further improve robustness, we applied the weighted median method, which can yield consistent estimates even when up to 50% of the instruments are invalid. Heterogeneity among SNP-specific causal estimates was assessed using Cochran Q test. We also implemented MR-PRESSO to identify and remove outlier SNPs that may introduce bias. Additionally, leave-one-out sensitivity analyses were conducted to evaluate the influence of individual SNPs on the overall estimate, and both scatter plots and funnel plots were generated to visualize potential pleiotropy and heterogeneity.

**Figure 2.**
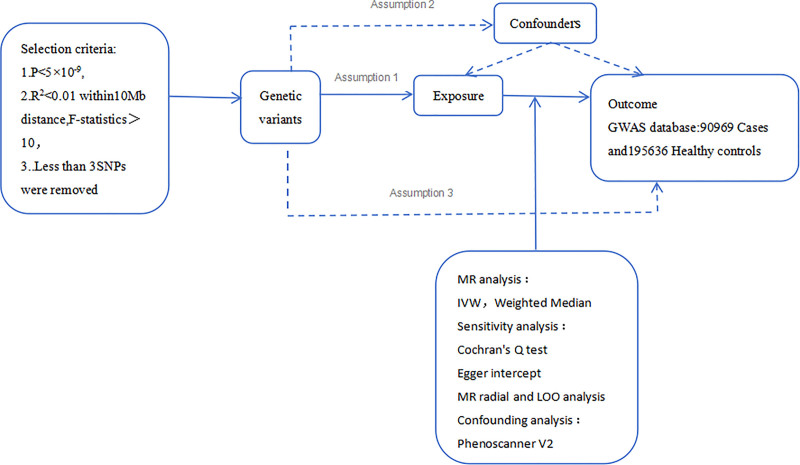
Overview of the overall MR design. Assumption 1, instrument variables are robustly related to exposure; Assumption 2, instrument variables are not related to confounders; Assumption 3, instrument variables are related to outcome only through exposure. IVW = inverse-variance weighted, LD = linkage disequilibrium, LOO = leave-one-out, MR = Mendelian randomization, SNPs = single-nucleotide polymorphisms.

Together, these complementary approaches formed a comprehensive and robust MR analytical framework that allowed for triangulation of evidence, reduced susceptibility to bias, and enhanced the credibility of our causal inferences(Fig. 2).Additionally, leave-one-out sensitivity analyses were performed to evaluate the influence of each SNP, and representative scatter and funnel plots (Figure 5 and Figure 6) have been added to visualize potential pleiotropy and heterogeneity.

**Figure 3.**
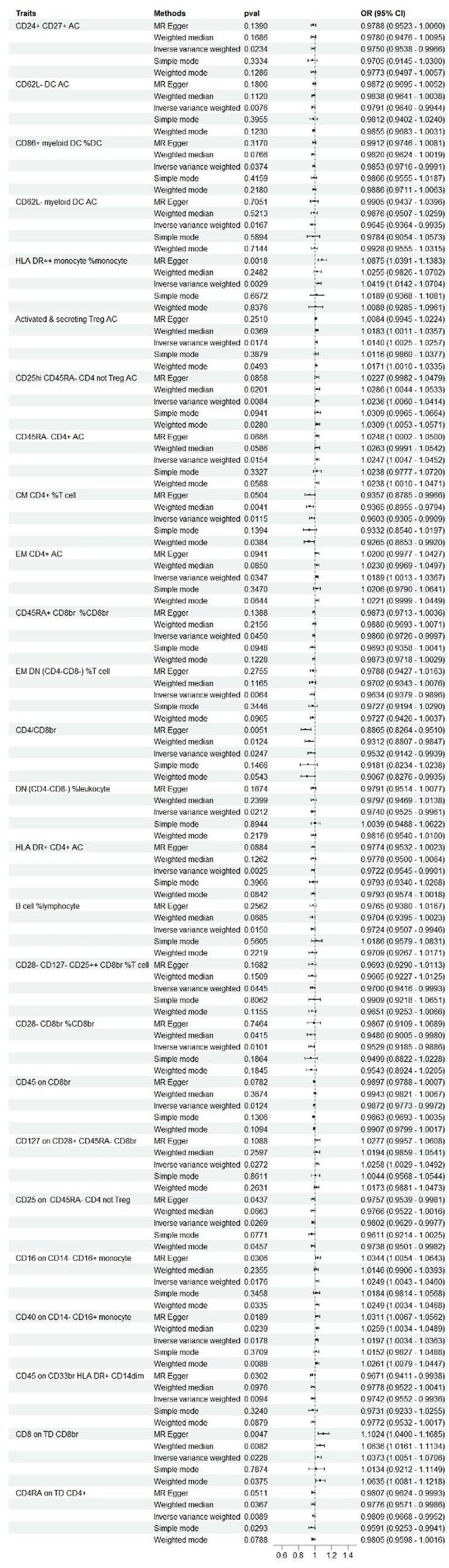
Mendelian randomization estimates of the association between immune cells and risk of breast cancer.

**Figure 4.**
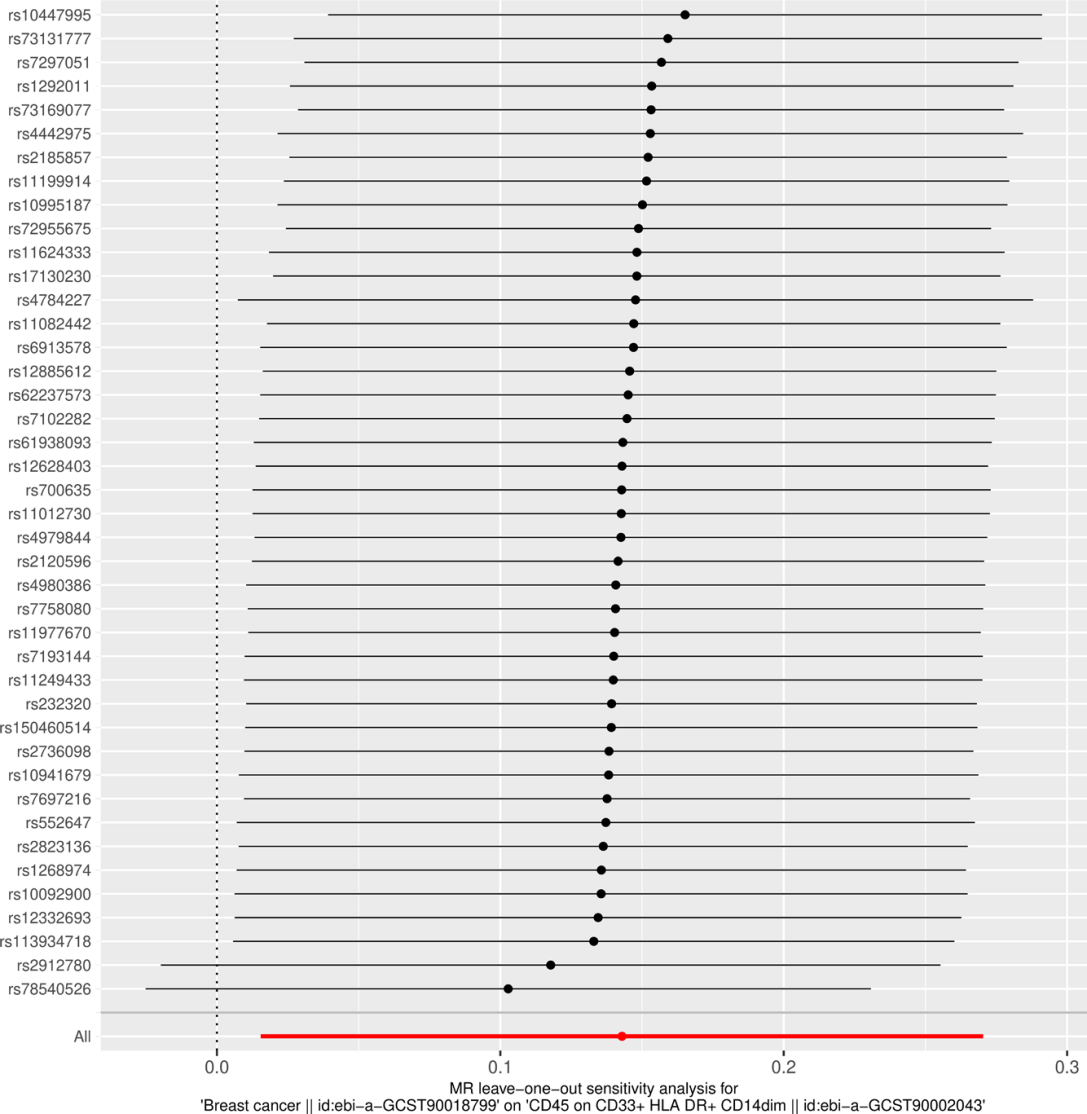
Leave-one-out sensitivity analysis for the association between genetically predicted CD45⁺CD33⁺HLA-DR⁺CD14^dim monocytes and breast cancer risk.

### 2.3. Heterogeneity and sensitivity analysis

We assessed heterogeneity across SNP-specific causal estimates using Cochran Q statistic and the I² index. Substantial heterogeneity was indicated by an I² > 50% or a Q test *P*-value <.05. In such cases, we applied the inverse-variance weighted (IVW) method under a random-effects model to accommodate between-SNP variability and provide more conservative causal estimates.

Leave-one-out sensitivity analyses were also visualized in scatter and funnel plots, demonstrating that no single SNP unduly influenced the overall results and that no strong evidence of directional pleiotropy was present. To evaluate the robustness of our findings, we compared results across multiple MR methods, including IVW, weighted median, and MR-Egger regression. Consistency among these methods strengthened the credibility of the causal inferences by triangulating evidence from different modeling assumptions.

We also performed leave-one-out sensitivity analyses, in which each SNP was iteratively excluded to assess its individual influence on the overall causal estimate. SNPs that significantly altered the results upon removal were further investigated for biological plausibility and heterogeneity contribution, and excluded when appropriate.

In cases of notable heterogeneity, we examined the directionality and magnitude of SNP-specific estimates and decomposed heterogeneity to identify whether a small number of outlier variants disproportionately contributed to the overall variance.

These layered sensitivity analyses established a systematic and resilient analytical framework, substantially enhancing the interpretability and credibility of our causal conclusions.

### 2.4. Data collection

We assessed the causal relationships between 731 immune cell traits and BC using MR analysis. These traits spanned 4 major categories: median fluorescence intensity (n = 389), absolute cell (AC) counts, relative cell counts, and morphologic parameters. The first 3 categories covered various immune subsets, including myeloid cells, B cells, monocytes, and T cells at different maturation stages. Panels such as TBNK (T cells, B cells, NK cells) and circulating dendritic cells (DCs) were included across both AC and morphologic parameters categories. Genetic instruments were derived from summary statistics of a large-scale GWAS of blood cell traits conducted by the Blood Cell Consortium (BCX), providing comprehensive genotype-phenotype associations relevant to hematologic and immune phenotypes.

### 2.5. Statistical analysis

The data analysis process was mainly done in R software version 4.0.2 (http://www.Rproject.org). In order to assess the causal relationship between 731 immunophenotypes and BC, IVW, weighted median and simple pattern analysis were performed mainly using the “MendelianRandomization” package (version 0.4.3). Heterogeneity among the selected IV was tested by Cochran Q statistic and its corresponding *P*-value by.^[19–21]^ If the original hypothesis was rejected, a random-effects inverse-variance weighted (IVW) approach was used to replace the fixed effects IVW approach.^[22]^ The explained strength of the selected SNPs was calculated by PVE (phenotypic explained variance) with the formula: PVE = 2 × EAF × (1-EAF) × β2. Next, the F-statistic was used to assess the strength of the IV, which reflects the exposed variance explained by the IV. The *F*-statistic was calculated based on the values of the PVEs, with the formula: [PVE × (n - 1 - k)}/[(1 - PVE) × k]. (EAF denotes effect allele frequency, β denotes effect size on exposure, n is the effective sample size of exposed genome-wide association studies (GWAS), and k is the number of variants included in the IV model). The statistical power of the MR analysis was estimated using an online calculator (https://shiny.cnsgenomics.com/mRnd/) with a type I error rate of α = 0.05 and the odds ratio from the IVW estimates. To rule out the effect of horizontal pleiotropy, the MR-Egger method was used, which states that horizontal pleiotropy is indicated if its intercept term is statistically significant. In addition, funnel plots and scatter plots were used to assess the robustness and heterogeneity of the correlations and whether the results were affected by outliers.

## 3. Results

### 3.1. Causal effects of immune phenotypes on BC

In our 2-sample MR analysis, we applied the inverse-variance weighted (IVW) method as the primary approach to investigate the causal effects of various circulating immune phenotypes on BC risk.^[23]^ Our findings revealed that several immune phenotypes were significantly associated with reduced BC risk. Notably, a number of traits demonstrated consistent protective associations, including CD24⁺CD27⁺ activated cells (AC), CD62L⁻ DC, CD86⁺ myeloid DCs, CD62L⁻ myeloid DC AC, CD28⁻CD127⁻CD25⁺⁺CD8^bright T cells, CD45RA⁺CD8^bright T cells, EM double-negative (CD4⁻CD8⁻) T cells, CD4/CD8^bright ratio, DN (CD4⁻CD8⁻) % leukocytes, HLA-DR⁺CD4⁺ AC, B cells as % of lymphocytes, CD28⁻CD8^bright % CD8^bright, CD45 on CD8^bright, CD25 on CD45RA⁻CD4⁺ non-Tregs, CD45 on CD33^bright HLA-DR⁺CD14^dim monocytes, and CD4RA on terminally differentiated (TD) CD4⁺ T cells.

Specifically, the proportion of CD4RA⁺ TD CD4⁺ lymphocytes was associated with a significantly reduced risk of BC (OR = 0.9809, 95% CI: 0.9668–0.9952, *P* = .0089), as determined by IVW analysis. This protective effect was consistent across additional MR methods, including the weighted median (OR = 0.9776, 95% CI: 0.9571–0.9986, *P* = .0367) and simple mode (OR = 0.9591, 95% CI: 0.9253–0.9941, *P* = .0293) approaches. In contrast, the weighted mode method did not yield statistically significant associations (OR = 0.9805, 95% CI: 0.9598–1.0016, *P* = .0788).

We further clarified the methodological assumptions underlying each MR approach. The IVW method provides the most powerful estimates under the assumption that all instruments are valid. The weighted median approach allows for up to 50% invalid instruments while still generating unbiased estimates. The simple and weighted mode methods rely on the majority-of-valid-instruments assumption; the latter incorporates SNP-specific weights to improve precision but is more sensitive to heterogeneity and bias. The lack of statistical significance in the weighted mode analysis may reflect its heightened sensitivity to pleiotropy and directional inconsistency. Rather than undermining our findings, this result highlights the complementary nature of multiple MR methods and underscores the robustness of the overall causal inference.

Additionally, we focused on the potential biological relevance of CD45RA expression on TD CD4⁺ T cells in BC development. Figure 3 summarizes the causal estimates of 731 immune phenotypes with respect to BC risk. Traits with ORs < 1 (e.g., CD4RA⁺ TD CD4⁺ T cells, CD28⁻CD127⁻CD25⁺⁺CD8⁺ T cells, B cells as % of lymphocytes) were associated with protection, while those with ORs > 1 (e.g., HLA-DR⁺ monocytes, activated Tregs, EM CD4⁺ T cells) indicated elevated risk. Significance thresholds and effect directions were clearly annotated in the figure for interpretability.

### 3.2. Causal effects of BC on immune phenotypes

We also performed a reverse-direction 2-sample MR analysis to assess the causal effects of BC on immune cell phenotypes. Multiple testing correction was applied using the false discovery rate method. Using the IVW approach, we identified significant associations between BC and the abundance of several immune cell populations. These included elevated levels of HLA-DR⁺ monocytes, activated and secreting Tregs, CD25^hi CD45RA⁻CD4⁺ non-Tregs, CD45RA⁻CD4⁺ AC, EM CD4⁺ AC, CD127 on CD28⁺CD45RA⁻CD8^bright T cells, CD16 on CD14⁻CD16⁺ monocytes, CD40 on CD14⁻CD16⁺ monocytes, and CD8 on TD CD8^bright T cells (see Table 1 for details).

**Table 1:** Reverse Mendelian randomization analysis of causal effects of breast cancer on immune phenotypes (IVW estimates).

Immune phenotype	OR (IVW)	95% CI	*P*-value
HLA-DR⁺ monocytes	1.073	1.023–1.125	.0025
Activated & secreting Tregs	1.075	1.012–1.141	.0174
CD25^hi CD45RA⁻ CD4⁺ non-Tregs	1.073	1.018–1.131	.0084
CD45RA⁻ CD4⁺ AC	1.026	1.004–1.049	.0154
EM CD4⁺ AC	1.038	1.003–1.074	.0347
CD127 on CD28⁺ CD45RA⁻ CD8^bright T cells	1.074	1.014–1.137	.0130
CD16 on CD14⁻ CD16⁺ monocytes	1.044	1.007–1.082	.0176
CD40 on CD14⁻ CD16⁺ monocytes	1.063	1.023–1.104	.0018
CD8 on TD CD8^bright T cells	1.122	1.021–1.234	.0177

CI = confidence interval, IVW = inverse-variance weighted, OR = odds ratio.

Only IVW results are presented, extracted from the MR analysis results. Significant associations were identified after controlling for multiple testing using the FDR method.

In contrast, several other immune phenotypes displayed significantly reduced expression levels in BC patients (*P* < .05), as illustrated in Figure 3. These findings suggest that altered immune cell composition may play a pivotal role in the etiology and progression of BC. However, this hypothesis requires further validation in large-scale clinical studies.

### 3.3. Specific immune phenotypes increase BC risk

**Figure 5.**
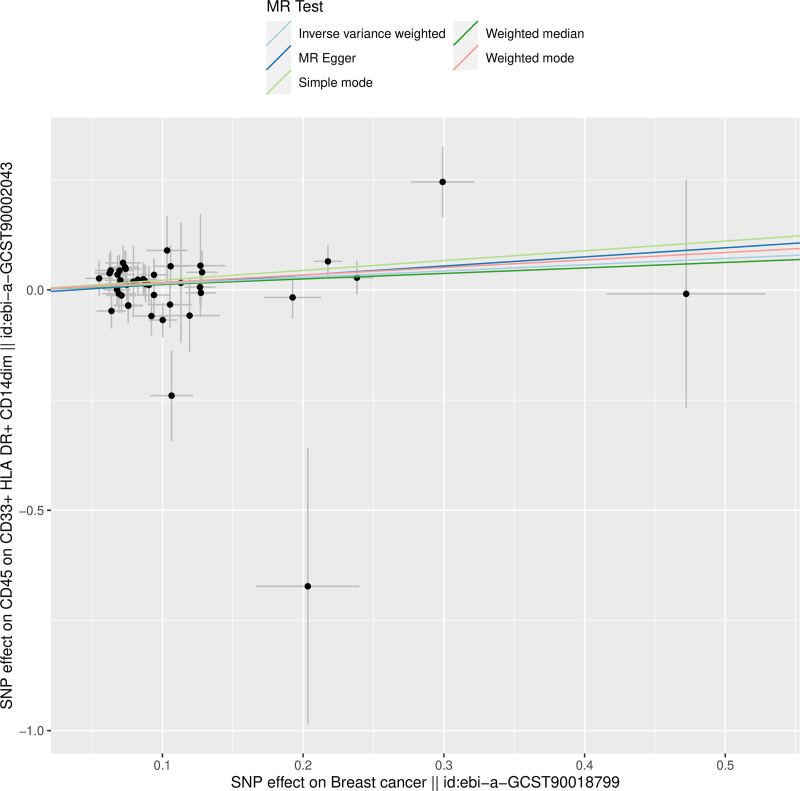
Scatter plot of the MR analysis. MR = Mendelian randomization.

**Figure 6.**
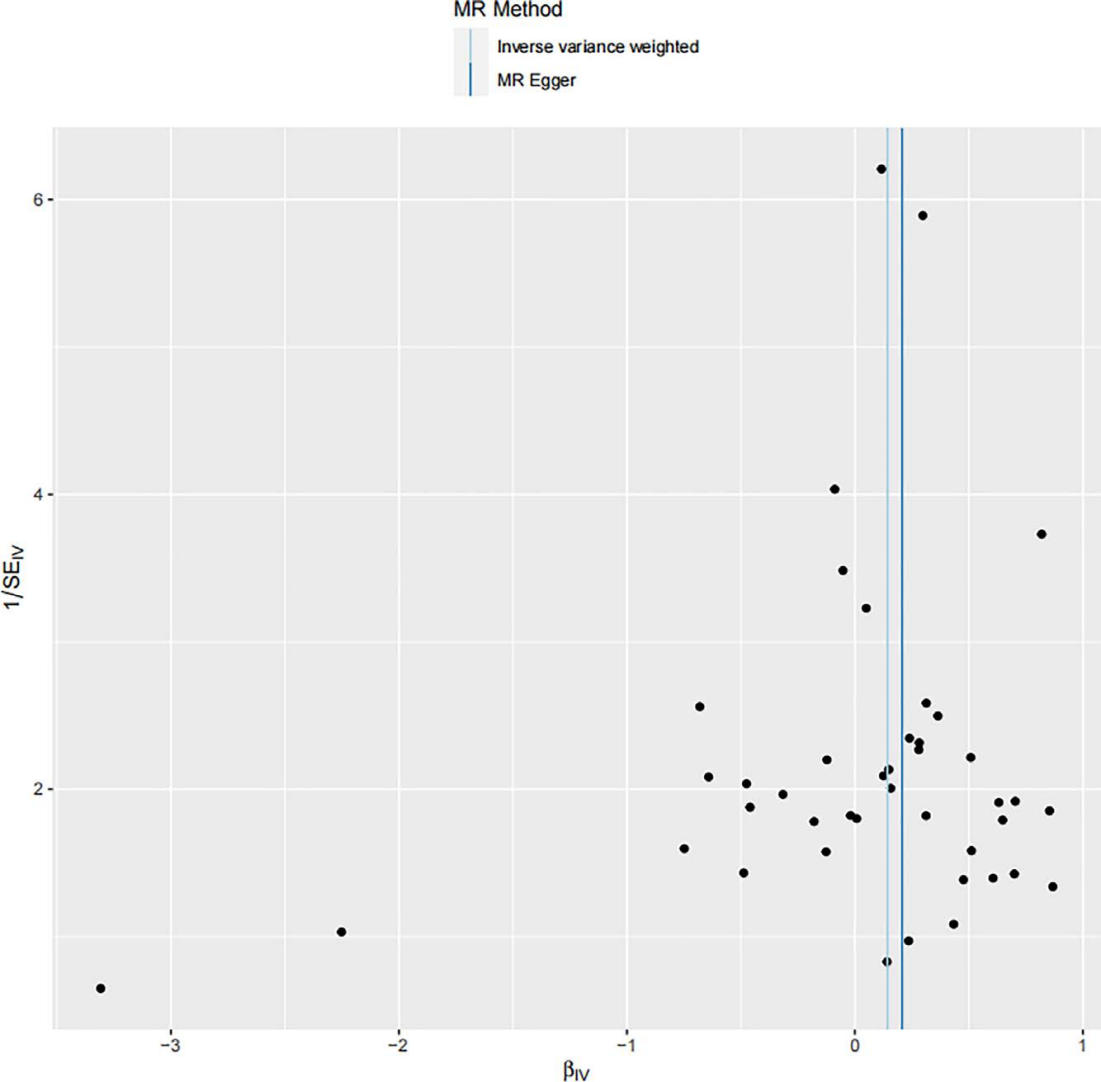
Funnel plot of the MR analysis for genetically predicted CD45⁺CD33⁺HLA-DR⁺CD14^dim monocytes and breast cancer risk. MR = Mendelian randomization.

To further evaluate the robustness and validity of the causal association, sensitivity analyses were performed. The leave-one-out analysis (Fig. 4) demonstrated that the exclusion of any single SNP did not materially alter the overall causal estimate, suggesting that the findings were not driven by a single genetic variant. The scatter plot (Fig. 5) illustrated the SNP-specific effects and regression slopes for multiple MR methods, confirming the consistency and direction of the association. Moreover, the funnel plot (Fig. 6) showed a symmetrical distribution of SNP-specific estimates, indicating no substantial directional pleiotropy and supporting the robustness of the causal inference.

**Figure 7.**
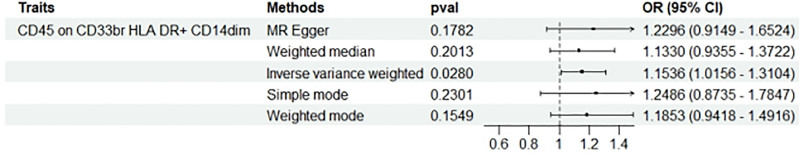
Mendelian randomization estimates the significant association between immune cells and the risk of breast cancer.

In these MR analyses, the association between CD45 expression in cells with high CD33 brightness, HLA-DR positivity, and low CD14 expression and BC risk was evaluated using multiple MR methods. Although the MR-Egger, weighted median, simple mode, and weighted mode methods did not yield statistically significant associations, the IVW method demonstrated a significant causal estimate for CD45 expression on CD33⁺HLA-DR⁺CD14^dim cells (*P* = .028, odds ratio = 1.1536; Figure 7). This finding underscores the analytical sensitivity of this phenotype’s causal estimate and highlights the robustness of the IVW-based results.

## 4. Discussion

In this study, we leveraged large-scale GWAS data to systematically evaluate the causal effects of 731 immune phenotypes on BC risk using a MR framework. This approach effectively minimized confounding and reverse causality. We identified 53 immune traits with significant associations, among which 7 remained statistically significant after Bonferroni correction, suggesting a potentially pivotal role in BC pathogenesis. These included CD24⁺CD27⁺ regulatory/memory B cells, CD62L⁻ and CD86⁺ mature dendritic cells (DCs), activated/naive CD8⁺ T cells, double-negative T cells (likely γδ T cells), HLA-DR⁺ CD4⁺ T cells, and TD CD4⁺ TEMwhich are known to participate in antigen presentation, immune memory maintenance, and RA cells, all of TME remodeling.

Building on these findings, we next compared our results with previous MR studies on immune cell traits in BC. Our findings align with previous MR studies in this field. For example, Xu et al (2024) leveraged a 2-sample MR combined with meta-analysis of BCAC and FinnGen GWAS data and identified 2 protective immune cell phenotypes – double-negative (CD4^–CD8^–) T cells and HLA-DR^+ immune cells – significantly associated with lower BC risk.^[12]^ Notably, we also observed these associations, with double-negative T cells and HLA-DR^+ CD4^+ T cells emerging among our top findings, reinforcing evidence for their protective role. At the same time, our broader screening uncovered additional immune-related traits, including specific B-cell and dendritic cell subsets, that were not highlighted by Xu et al,^[12]^ demonstrating the novel insights gained from our comprehensive analysis. Furthermore, while Xu et al reported no reverse causal effect of BC on those immune traits (consistent with our reverse MR results), an earlier MR by Wu et al (2025) suggested bidirectional influences, identifying 27 immune cell traits associated with BC risk and noting that BC might causally affect certain immune cell levels.^[8]^ The difference between Wu et al bidirectional findings and our predominantly unidirectional results may reflect variations in statistical power, analytical thresholds, or population heterogeneity. Overall, by contextualizing our findings alongside these prior studies, we clarify that our investigation builds upon existing evidence – rather than representing the first MR-based study of immune cells in BC – and adds significant breadth and depth to the understanding of how diverse immune cell populations may causally influence breast oncogenesis.

Of particular interest, we highlighted the potential protective role of CD45RA expression on TD CD4⁺ T cells. CD45RA is a hallmark of naive CD4⁺ T cells, representing a pool of antigen-inexperienced yet antigen-responsive cells capable of rapid activation upon first exposure. This subset plays a critical role in immunosurveillance by clearing early tumor-initiating cells and has been associated with favorable outcomes in multiple solid tumors, supporting its candidacy as a protective factor.^[12]^ Other immune phenotypes showing protective trends, such as CD28⁻CD127⁻CD25⁺⁺CD8⁺ T cells and B-cell subsets, may also contribute to antitumor immunity by modulating the immune microenvironment.

Our reverse-direction MR analysis found no evidence of a significant genetic causal effect of BC on immune phenotypes (all *P* > .05), supporting a unidirectional relationship from immune phenotypes to BC. Several factors may account for this asymmetry: (1) the molecular heterogeneity of BC may dilute overall effects; (2) peripheral immune traits captured by GWAS do not reflect intratumoral immune dynamics; and (3) a limited number of instrumental SNPs may have constrained statistical power. The consistency between forward and reverse analyses enhances the robustness of our causal interpretation.

Previous studies have underscored the central regulatory role of immune cells in carcinogenesis, acting as key mediators between chronic inflammation and tumor progression.^[24,25]^ In BC, tumor evolution is tightly linked to immune cell composition. Regulatory T cells (Tregs), while essential for maintaining immune tolerance, may also suppress antitumor responses and facilitate tumor growth. Their enrichment in the TME has been associated with poor prognosis.^[26]^ MDSCs inhibit T and NK cell functions, promote immune escape, and are strongly implicated in BC progression.^[27–30]^

Minna Mutka et al observed reductions in CD3⁺, CD8⁺, and CD56⁺ cells during BC recurrence, with no significant changes in CD4⁺ cells.^[31]^ Subsets of CD4⁺ T cells can eliminate MHC class II-positive tumor cells directly or mediate the clearance of MHC class II-deficient tumor cells via myeloid cell recruitment.^[31]^ The chemokine CX3CL1 has been shown to recruit antitumor immune cells to the TME and suppress tumor growth.^[32]^ Some studies have proposed CD14⁺CD16⁺ monocytes as potential early detection markers for BC, but we found no significant causal association with BC risk in our MR analysis.^[33]^

Our findings were validated by multiple sensitivity analyses and were not meaningfully influenced by pleiotropy or confounding. However, several limitations remain. First, the current study did not examine causal relationships between immune phenotypes and other cancers (e.g., lung, thyroid, colorectal), limiting generalizability. Second, GWAS data were predominantly derived from individuals of European ancestry, which restricts trans-ethnic applicability. Populations differ in immunogenetic backgrounds, tumor biology, and environmental exposures; for example, Asian populations exhibit distinct patterns in age of onset, molecular subtypes, and immune profiles. Future studies should incorporate GWAS data from Asian and multi-ethnic cohorts to conduct population-specific or cross-ancestry MR analyses.

Despite our use of MR-Egger regression, weighted median, MR-PRESSO, and leave-one-out methods to rigorously evaluate MR assumptions, potential biases cannot be fully excluded: some IV had low F-statistics, indicating possible weak instrument bias; horizontal pleiotropy may violate the InSIDE assumption; mismatches between genotype and phenotype measurements; and residual confounding. Future work should adopt stricter IV selection criteria, increase sample sizes, explore multivariable and Bayesian MR methods, and integrate transcriptomic, proteomic, and epigenomic data to enhance causal inference at multiple mechanistic levels.

Immune cells play a critical role in BC initiation and progression, with important implications for both research and clinical practice. Elucidating the pathogenic mechanisms of immune phenotypes may inform risk stratification, immune subtyping, and targeted intervention strategies. Although immunotherapy is now widely used across cancer types, patient selection and response prediction remain key challenges. Specific immune phenotypes may serve as predictive markers for BC risk and aid in individualized treatment decision-making. Integrating peripheral immune profiling, inflammatory markers, PD-1/PD-L1 expression, and tumor-infiltrating lymphocyte (TIL) status could improve early detection and enhance responsiveness to immunotherapy.

## 5. Conclusion

Based on large-scale GWAS data, this study employed bidirectional MR to elucidate direct causal relationships between multiple immune phenotypes and BC risk, effectively minimizing confounding and reverse causality. Several key immune traits – such as CD24⁺CD27⁺ B cells, CD28⁻CD127⁻CD25⁺⁺ CD8⁺ T cells, and TD effector memory T cells (TEMRA) – exhibited consistent protective or risk effects, underscoring their potential as biomarkers for screening and targets for immunotherapy. Integration with liquid biopsy, immune scoring, and TIL characteristics may enable the development of risk prediction models and personalized treatment strategies for BC.

Although our findings were robust, validation in multi-ethnic populations is warranted. Experimental studies are also needed to accelerate the clinical translation of immune phenotypes. Future work should incorporate integrative multi-omics approaches and longitudinal data to refine immune-based subtyping and advance precision intervention strategies in BC.

## Author contributions

**Conceptualization:** Ning Zhai.

**Data curation:** Jinxin Bai.

**Investigation:** Wenwen Luo, Dequan Pang.

**Methodology:** Duo Han, Dequan Pang.

**Software:** Chunhui Liu.

**Validation:** Wenzi Luo.

**Visualization:** Wenzi Luo.

**Writing – original draft:** Wenzi Luo.
